# Vitamin C biofortification of broccoli microgreens and resulting effects on nutrient composition

**DOI:** 10.3389/fpls.2023.1145992

**Published:** 2023-03-03

**Authors:** Shivani Kathi, Haydee Laza, Sukhbir Singh, Leslie Thompson, Wei Li, Catherine Simpson

**Affiliations:** ^1^ Department of Plant and Soil Science, Texas Tech University, Lubbock, TX, United States; ^2^ Department of Animal and Food Sciences, Texas Tech University, Lubbock, TX, United States; ^3^ Department of Chemical Engineering, Texas Tech University, Lubbock, TX, United States

**Keywords:** ascorbic acid, brassica, microgreens, biofortification, vitamins, malnutrition

## Abstract

The consumption of plants plays an important role in human health. In addition to providing macro and micronutrients, plants are the sole sources of several phytonutrients that play a major role in disease prevention. However, in modern diets, increased consumption of cheaper, processed foods with poor nutritional value over fruits and vegetables leads to insufficient consumption of essential nutrients such as vitamin C. Taking supplements can address some of the insufficient nutrients in a diet. However, supplements are not as diverse or bioavailable as the nutrients in plants. Improving the abundance of nutrients in plants will reduce the amounts that need to be consumed, thereby reducing the price barrier and use of supplements. In this study, broccoli (*Brassica oleracea* var. *italica*) microgreens grown in a controlled environment were biofortified for increased vitamin C content. The microgreens grown on growing pads were treated with supplemental nutrient solutions. Treatments were applied four to five days after germination and included four different concentrations of ascorbic acid specifically, 0% (control), 0.05%, 0.1%, 0.25% and 0.5%, added to the nutrient solution. Microgreens with turgid cotyledons and appearance of tip of first true leaves were harvested about 14 days after germination and were analyzed for biomass, chlorophylls, carotenoids, vitamin C and other minerals content. The ascorbic acid improved the microgreens’ fresh biomass, percent dry matter, chlorophylls, carotenoids, vitamin C, and potassium content. Moreover, this study also mapped out the correlation between ascorbic acid, phytochemicals, and broccoli microgreens’ mineral composition. The total vitamin C was positively correlated to K and negatively correlated to chlorophylls, N, P, Mg, Ca, S, and B (p < 0.01). These relationships can be applied in future vitamin C biofortification research across different microgreens. In conclusion, vitamin C was increased up to 222% by supplemental ascorbic acid without being detrimental to plant health and mineral composition.

## 1. Introduction

Plants are fundamental sources of diverse essential macro and micronutrients, minerals, vitamins, and fiber. Plants also contain phytonutrients such as polyphenols, carotenoids, plant sterols and polyunsaturated fatty acids that aid in disease prevention and cannot be found in meat or dairy products (Martin and Li, 2017). Diversifying diets and consuming more fresh fruit and vegetables can improve human health and help combat malnutrition/hidden hunger, and other chronic diseases (Bradbury et al., 2014; Mena and Angelino, 2020). However, increasing costs of fruits and vegetables and the increased availability of cheaper processed foods are reducing consumption of healthy foods and affecting overall nutritional value of modern, Western diets (Martin and Li, 2017). These concerns call for a sustainable solution to help overcome malnutrition, undernutrition, and reduce the necessity for supplements.

Food insecurity has been increasing; where globally one in every ten people are malnourished (~ 10% of the global population) (Payne, 2021). There has been an increasing need to improve the yield and nutritional composition of plants to feed the growing population. However, producing more with less to have enough for this population affects produce quality. Davis (2009) reported the “dilution effect,” where the yield is inversely related to mineral composition of plants due to environmental effects and breeding. To address these issues, improving nutritional quality of produce while not affecting the yield of the produce is necessary. Biofortifying plants increases the nutrient content in plants and results in improved nutrient profiles (Sharma et al., 2017). However, agronomic biofortification has advantages over breeding and biotechnology methods of biofortification due to the time and public concerns about GMOs that these techniques involve (Singh et al., 2016; Garg et al., 2018; Prasad and Shivay, 2020). Agronomic biofortification in horticultural crops grown in controlled environments allows for relatively easier control and manipulation of crops than that of field grown crops because of scale and high value of plants produced therein. Agronomic biofortification results in nutrient dense crops that, upon consumption, can alleviate deficiencies and be a healthy and reliable way to maintain human health (Joy et al., 2014; de Valença et al., 2017).

Microgreens are a versatile crop and can be grown on a small or large scale, yet have higher concentrations of nutrients compared to their mature forms (Mir et al., 2017). This makes them excellent candidates for biofortification studies. Several studies have attempted to improve microgreen nutrient profiles with success. However, these attempts were limited to few nutrients such as iron (Fe), zinc (Zn), selenium (Se) and iodine (I) (Di Gioia et al., 2019; Germ et al., 2019; Newman et al., 2019; Puccinelli et al., 2019; Pannico et al., 2020; Newman et al., 2021; Puccinelli et al., 2021) and few phytochemicals such as carotenoids (Brazaityte et al., 2015; Samuolienė et al., 2017). It is important to extend these biofortification attempts across all the essential nutrients, especially vitamins.

Vitamins are significantly harder to biofortify due to their complex biosynthesis pathways and instability (Raiola et al., 2015; Paciolla et al., 2019). Specifically, vitamin C (Vit C) is an antioxidant not produced in humans and needs to be consumed *via* plants (Padayatty and Levine, 2016). Vit C deficiencies can cause nonspecific symptoms such as fatigue, apathy, anxiety and depression in elderly populations making it difficult to diagnose (Baradhi et al., 2018). However, due to changes in quality of produce and growing methods, the supply is often inconsistent. Hence, it is important to biofortify Vit C to provide a uniform supply while not compromising other nutrients. Efforts to biofortify Vit C have primarily been attempted through breeding and biotechnology as reported by Paciolla et al. (2019). This is likely because the increase of Vit C using agronomic methods is unstable and disappears at the time of harvest or after 24-72h in mature plants (Havas, 1935; Freebairn and Taylor, 1960; Freebairn, 1963; Mozafar and Oertli, 1993). However, microgreens were not a popular crop when many of these studies took place, which could be the reason for few biofortification attempts in young plants. Hausen (1935) reported the possibility of biofortifying Vit C in early stages of seedling growth. Only four studies (De Souza et al., 2019; Garcia Neto et al., 2021; dos Santos et al., 2022; Kathi et al., 2022) were found in the past decade that had successfully increased Vit C content in horticultural crops. of these, there were fewer studies that document how this biofortification occurs in microgreens (Kathi et al., 2022). In fact, Kathi et al. (2022) found that arugula microgreens can be biofortified with exogenous applications of ascorbic acid. However, this study was limited to arugula (*Eruca sativa ‘Astro’*) microgreens and did not document the relationship between Vit C biofortification and other nutrients content. Because each crop is unique in their composition, evaluating various microgreens is essential to determine the differences in effects of ascorbic acid application. Thus, the objectives of this study were to determine the impacts of ascorbic acid application on broccoli microgreens growth, mineral nutrients and phytochemicals content.

## 2. Materials and methods

### 2.1. Plant growth and harvest

Commercially produced broccoli seeds were purchased from Easy Peasy Plants (Alvin, Illinois, USA). Seeds were grown on a growing pad (Micromat, Salt Lake City, Utah, USA) at a rate of 5 g in 127 X 127 mm lidded trays (Weber, 2017). The seeds were misted with deionized (DI) water to facilitate germination. The growing pads were kept moist with the application of DI water until the ascorbic acid (AA) treatments were applied. Five days after germination, at the appearance of cotyledonary leaves treatments were applied (as indicated in **Table 1**). Treatments consisted of 2-1-6 Floragro (NPK; General Hydroponics, Santa Rosa, California, USA) supplemented with 0% (control), 0.05%, 0.1%, 0.25% and 0.5% AA (L-(+)-Ascorbic acid, Alfa Aesar, Haverhill, Massachusetts, USA) with pH adjusted to be in between 6.0 - 6.5 with the addition of KOH and the electrical conductivity (EC) was also noted. The five treatments were replicated five times in a randomized block design in two different trials. Plants were grown at room temperature (approximately 22 °C) under supplemental LED lighting with the photosynthetically active radiation (PAR) of approximately 60-68 µmol m^-2^ s^-1^ for 12hr/day. The growing pads were fertigated with a known volume of treatment solutions to keep them moist (**Table 1**). Treatments began after the appearance of cotyledons and terminated at harvest. About 8-10 days after germination, the broccoli microgreens were harvested by cutting the shoot 2 mm above the surface of growing pad using sterilized stainless-steel scissors. The microgreens at harvest had turgid cotyledons and appearance of the tip of first true leaves (Mir et al., 2017; Weber, 2017). The growing pad and the tray were discarded and the fresh weight of the microgreens from each treatment was recorded. Following the biomass measurements, the labeled and bagged microgreens were stored at -80 °C, until further analysis. The frozen microgreens samples were then freeze-dried (HarvestRight, North Salt Lake City, Utah, USA) and the dry weight was recorded. The samples were then ground in the presence of liquid nitrogen and stored at -80°C.

**Table 1:** Amount of water and ascorbic acid (AA) applied to broccoli microgreens in each treatment grown in hydroponics system.

Trial	Treatment (% AA)	Electrical conductivity of the solution (µS cm^-2^)	Amount of treatment applied (mL) day^-1^ at each application										Total treatment volume applied (mL)	Total ascorbic acid applied (mg)	Total K applied (mg)
			1	2	3	4	5	6	7	8	9	10			
1	0	408	25	25	30	30	30	–	40	30	20	H	230	0	14
	0.05	651	25	25	30	30	30	–	40	30	20		230	115	43
	0.1	879	25	25	30	30	30	–	40	30	20		230	230	76
	0.25	1485	25	25	30	30	30	–	40	30	20		230	575	106
	0.5	2710	25	25	30	30	30	–	40	20	20		220	1100	251
2	0	411	40	30	30	30	30	30	30	30	H	–	250	0	15
	0.05	645	40	30	30	30	30	30	30	30			250	125	41
	0.1	910	40	30	30	30	30	30	30	30			250	250	84
	0.25	1563	40	30	30	30	30	30	30	30			250	625	111
	0.5	2520	40	30	30	30	30	30	0	30			220	1100	236

Numbers 1- 10 refer to the days after the appearance of cotyledonary leaves. Cells marked with ‘H’ indicate when microgreens were harvested.

### 2.2. Sample analysis

Chlorophylls (chl) and carotenoid analyses were performed according to Lichtenthaler (1987) with a few modifications to suit microplate spectrophotometry. In brief, 100 mg of ground microgreens were measured into 5-mL test tubes and then 1-mL 100% methanol was added to each sample. The resulting mixture was homogenized to facilitate extraction of chlorophylls and carotenoids and then subjected to centrifugation for 15 min at 10,000 rpm. The filtrate of the mixture (200 µL) was added to the microplate and absorbance was read and recorded at 665, 652, and 470 nm for chlorophyll a and b, and carotenoids, respectively with methanol as a blank using the microplate spectrophotometer (SpectroMax iD3, San Jose, California, USA).

Vitamin C extraction was done according to Sérino et al, (2019) and analysis as per Stevens et al, (2006). Briefly, Vit C was extracted by using 6% trichloroacetic acid. For total ascorbic acid or total Vit C (TAA), 20 µL of extractant of each sample was added to a microplate and reacted with dithiothreitol, N-ethyl maleimide and color reagent in that order and the absorbance was recorded at 550 nm. Simultaneously, for ascorbic acid content (AA), dithiothreitol and N- ethyl maleimide were replaced with phosphate buffer and the absorbance was recorded at 550 nm.

The ground microgreens were also analyzed for different minerals (N, P, K, Mg, Ca, S, B, Zn, Mn, Fe and Cu) by Waters Agricultural Laboratories, Inc. (Camilla, Georgia, USA).

### 2.3. *Estimated daily intake and* nutrient contribution *calculations*

Estimated daily intake and NC were calculated to quantify plant nutrition in relation to human intake. EDI provides the amount if Vit C obtained from the consumption of recommended size of microgreens whereas NC indicates the contribution of these microgreens to recommended daily Vit C consumption. Estimated daily intake and NC analysis were calculated according to Ghoora et al. (2020). FDA reference amount customarily consumed (RACC) used in the EDI calculation was that of mature broccoli (85g) since the RACC for microgreens was not available (FDA, 2016). Reference daily intake (RDI) used in the calculation of NC of Vit C for adults was 90 mg per day (FDA, 2020).

### 2.4. Statistical analysis

JMP Pro 16.0.0 (SAS Institute, Cary, North Carolina) was used to perform the statistical analysis. Factorial analysis and standard least squares regression models were performed and the significance among treatments within a trial were determined at P ≤ 0.05. since there were significant interactions between the trials for many variables that the data within each trial were analyzed separately. Tukey’s HSD or student’s t tests were used to differentiate means in statistically significant results. A multivariate correlation analysis was also performed to determine relationships between analyzed factors.

## 3. Results

### 3.1. Microgreen yields

Microgreens treated with AA had the highest fresh weight (wt.) in both trials compared to the control (**Table 2**). However, in trial 1, 0.05% AA treatment had greater fresh weight of the five different treatments followed by 0.1% AA treatment and in trial 2, 0.25% AA had the greatest fresh weight followed by 0.1% AA treatment. Furthermore, there were no statistically significant differences among the treatments in trial 1 in terms of dry weight but in trial 2, dry weight increased with AA application, where 0.5% AA-treated microgreens had the greatest dry weight. Similar results were seen with percent dry matter (%DM) as well, the higher AA rates had the greatest %DM. In trial 1, the 0.25% AA-treated microgreens had greater %DM due to plant mortality of 0.5% AA treated microgreens. Finally, in trial 2 the 0.5% AA treated microgreens and control treatments had higher %DM.

**Table 2:** Effects of ascorbic acid treatments on fresh weight, dry weight and % dry matter (% DM) of broccoli microgreens in trials 1 and 2.

	Trial-1			Trial-2		
Treatments (% AA*)	Fresh wt. (g)	Dry wt. (g)	%DM	Fresh wt. (g)	Dry wt. (g)	%DM
0	23.0 b	1.7	7.5 b	9.9 c	1.4 b	14.3 a
0.05	28.1 a	1.9	6.9 b	18.6 b	1.6 b	8.8 b
0.1	25.6 ab	2.0	7.9 b	24.7 a	2.0 ab	8.1 b
0.25	22.4 b	2.0	8.9 a	27.9 a	2.2 a	8.1 b
0.5	–	–	–	14.8 bc	2.4 a	16.3 a
P-value	0.0097	0.0767	<0.0001	<0.0001	0.0003	<0.0001

Values within the same column with different letters are significantly different at p ≤ 0.05. *AA- ascorbic acid.

### 3.2. Composition of microgreens

#### 3.2.1. Chlorophylls and carotenoids in broccoli microgreens

**Figure 1:**
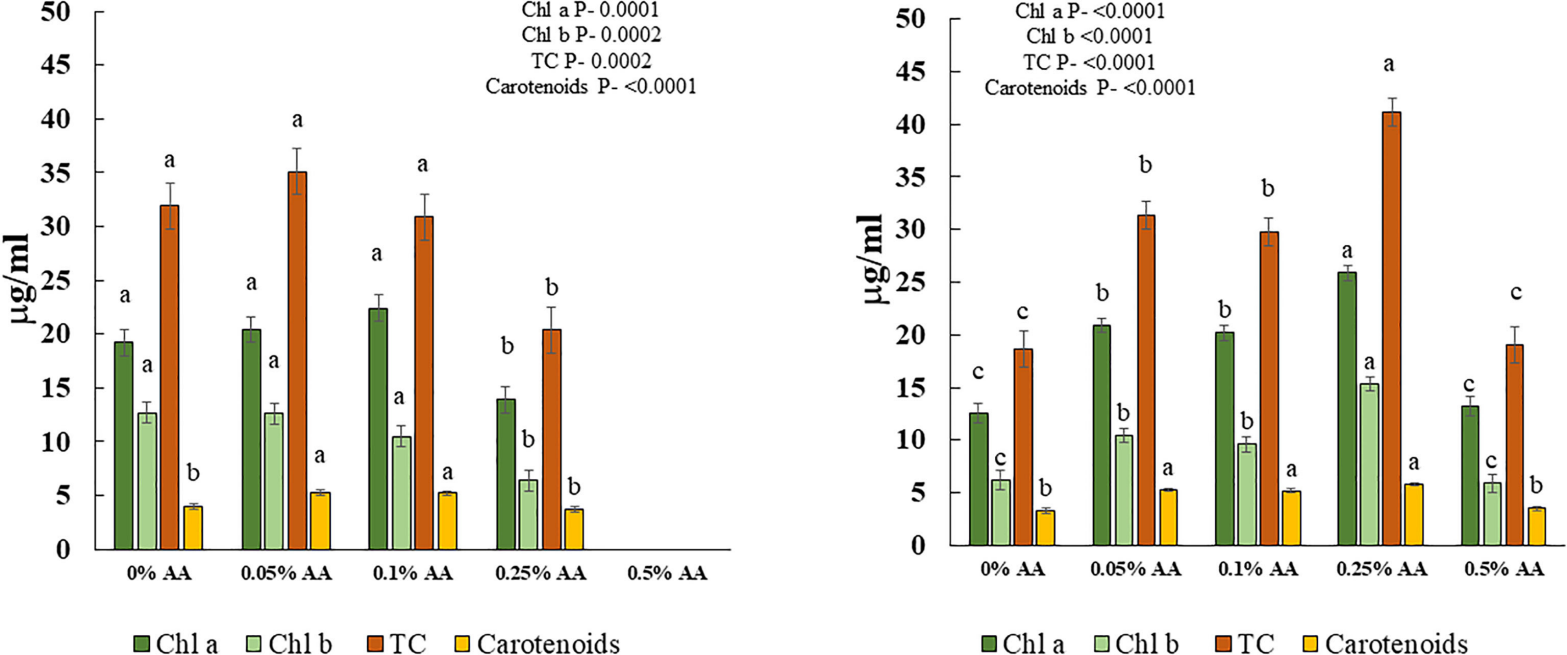
Effects of ascorbic acid treatments on chlorophyll a (chl a), chlorophyll b (chl b), total chlorophylls (TC) and carotenoids of broccoli microgreens in [Left panel] trial 1 and [Right panel] trial 2. Differences among means within each compound are indicated by different lowercase letters. Error bars represent ±1 standard error of the mean.

There was a significant effect of AA treatments on chl a & b, total chlorophylls (TC), and carotenoids in both trials (**Figures 1** left and right panels). Specifically, greater values of chl a & b, TC and carotenoids were seen in microgreens treated with lower concentrations of AA (i.e., 0.05% and 0.1%) in trial 1 and in 0.25% AA treatment in trial 2.

#### 3.2.2. Vitamin C and ascorbic acid in broccoli microgreens

**Figure 2:**
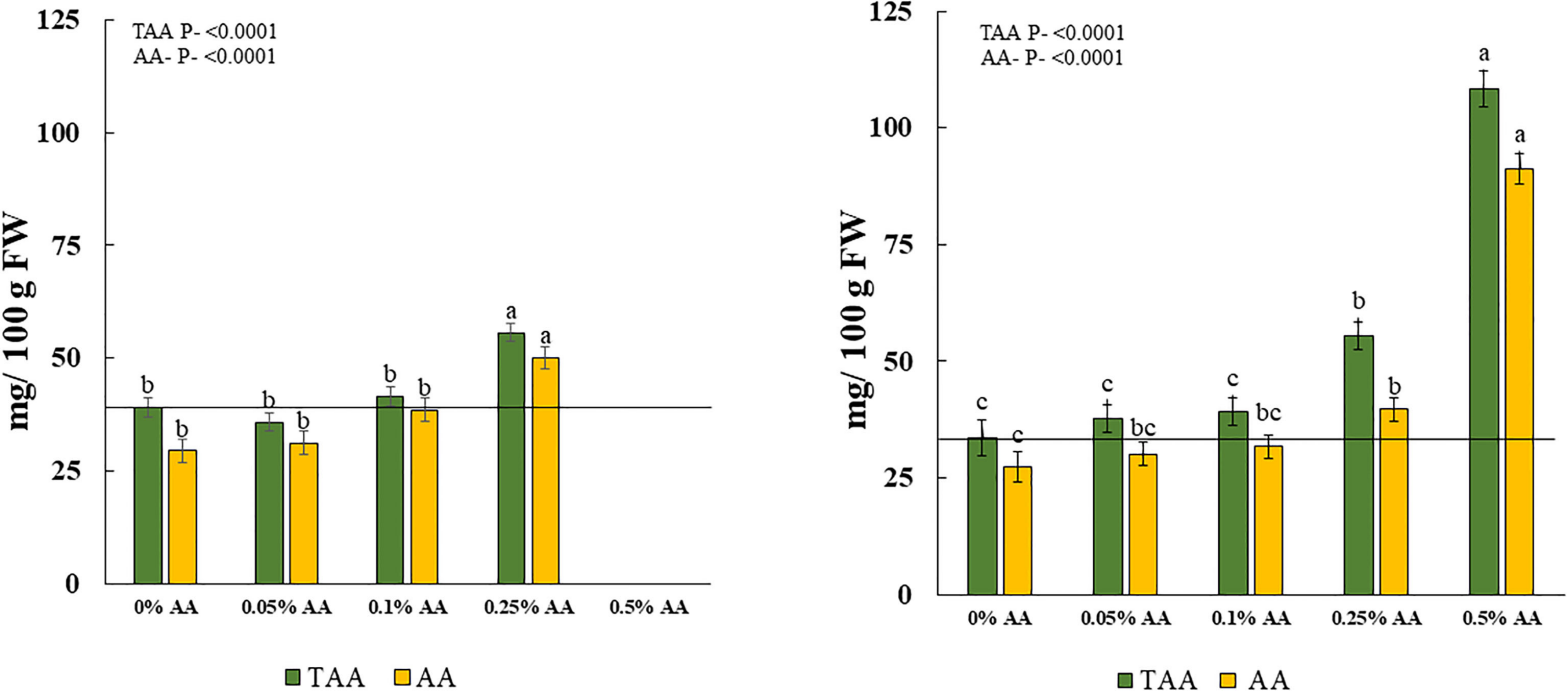
Effects of ascorbic acid treatments on total ascorbic acid/total vitamin C (TAA) and ascorbic acid (AA) content in fresh matter (FM) of broccoli microgreens in [Left panel] trial 1 and [Right panel] trial 2. Horizontal lines indicate average vitamin C in non-biofortified broccoli microgreens. Differences among means at p<0.05 with in treatments are shown by different lowercase letters within green or yellow bars. Error bars represent ±1 standard error of the mean.

The total Vit C or total ascorbic acid (TAA) and AA content in broccoli microgreens were significantly affected by the treatments in both trials. TAA increased with increased application of ascorbic acid from 0.1% AA to 0.5% AA. The highest amount of TAA and AA in fresh weight (FW) was seen in treatments of 0.25% AA followed by 0.1% AA treated microgreens in trial 1 and the greatest amount of TAA in FW was seen in 0.5% AA followed by 0.25% AA treated microgreens in trial 2. Furthermore, a similar pattern was observed for AA. The highest amount of AA was seen in 0.25% AA in trial 1 (**Figure 2** left panel) and 0.5% AA in trial 2 (**Figure 2** right panel). Even though, 0.5% AA treated microgreens did not survive in trial 1, they resulted in greater increases in both TAA and AA by 222% and 232.5%, respectively in trial 2. The 0.25% AA treated microgreens resulted in an average of 53.9% and 57.4% increase in TAA and AA, respectively (**Table 3**).

**Table 3:** Average percent change of total ascorbic acid/total Vit C (TAA) and ascorbic acid (AA) in fresh weight of each treatment treated with different concentrations of ascorbic acid over both trials.

Treatments % AA	Trial-1		Trial-2	
	% Increase in TAA mg/100 g FM*	% Increase in AA mg/100 g FM	% Increase in TAA mg/100 g FM	% Increase in AA mg/100 g FM
0	–	–	–	–
0.05	-8.2	6.1	12.5	10.2
0.1	6.1	30.8	16.9	15.8
0.25	42.5	69.8	65.2	44.9
0.5	–	–	222.0	232.5

*FM- fresh matter.

#### 3.2.3. Mineral composition of broccoli microgreens

The effects of different rates of ascorbic acid on the nutrient composition of broccoli microgreens are shown in **Table 4**. Significant differences were seen among the treatments in the concentration of several nutrients like N, P, K, Mg, Ca, S, B, Zn, Mn, Fe and Cu in broccoli microgreens in both trials (**Table 4**). Moreover, greater N, P, Ca, S, Zn, Mn, Fe, and Cu concentrations were seen using lower AA treatments and control. Greater K content was seen at 0.25% AA treatments in both the trials and greater B content was seen in 0.1% AA in trial 1 and 0.05% AA in trial 2.

**Table 4:** Effects of ascorbic acid (AA) treatments on nitrogen (N), phosphorus (P), potassium (K), magnesium (Mg), calcium (Ca), sulfur (S), boron (B), zinc (Zn), manganese (Mn), iron (Fe) and copper (Cu) concentration of broccoli microgreens in trials 1 and 2.

Trial-1	Treatments (% AA)	N %	P %	K %	Mg %	Ca %	S %	B ppm	Zn ppm	Mn ppm	Fe ppm	Cu ppm
	0	6.4 a	1.2 a	2.2 d	0.5 a	0.44 a	1.9 a	35.0 b	73.8 a	26.8 a	83.0 a	5.6 a
	0.05	6.1 ab	1.2 a	3.3 c	0.5 a	0.43 ab	1.9 a	31.4 b	73.0 a	25.8 ab	82.4 a	6.0 a
	0.1	5.8 bc	1.1 b	4.1 b	0.5 a	0.43 ab	1.9 a	48.4 a	68.2 ab	24.6 ab	78.8 ab	5.2 a
	0.25	5.4 c	1.0 c	5.7 a	0.4 b	0.40 b	1.7 b	23.6 c	63.8 b	24.0 b	74.2 b	4.2 b
	0.5	–	–	–	–	–	–	–	–	–	–	–
	P-value	0.0004	<0.0001	<0.0001	0.0001	0.0387	<0.0001	<0.0001	0.0006	0.0311	0.0013	0.0006
Trial-2	Treatments	N %	P %	K %	Mg %	Ca %	S %	B ppm	Zn ppm	Mn ppm	Fe ppm	Cu ppm
	0	5.9 a	1.20 a	1.3 d	0.5 a	0.41 a	1.8 a	29.0 b	73.0 a	27.3 a	93.0 a	6.7 a
	0.05	5.9 a	1.20 a	2.1 c	0.5 a	0.41 a	1.8 ab	34.6 a	71.0 a	25.8 ab	83.6 b	5.6 ab
	0.1	5.7 ab	1.10 ab	2.8 b	0.5 a	0.41 a	1.8 ab	29.6 b	70.6 a	25.0 b	83.4 b	5.6 ab
	0.25	5.5 b	1.00 c	3.8 a	0.4 b	0.39 b	1.6 c	23.6 c	65.0 b	24.7 b	76.8 c	4.6 b
	0.5	5.5 b	1.03 bc	3.2 ab	0.4 b	0.38 b	1.7 bc	19.7 c	62.8 b	22.4 c	84.3 b	4.7 b
	P-value	0.0028	0.0005	<0.0001	<0.0001	0.0257	0.0418	<0.0001	<0.0001	<0.0001	0.0002	0.0009

Values within the same column with different letters are significantly different at p ≤ 0.05.

Correlation coefficients (below diagonal) among composition attributes such as chl a and b, total chls, carotenoids, TAA and AA in fresh matter and different nutrients in broccoli microgreens grown under different concentrations of AA are shown in **Table 5**. And, It was observed that there was strong relationship between TAA and AA which were highly positively associated (p<0.0001), as expected (**Table 5**). Next, TAA also had strong inverse relationships with chl a and b, carotenoids, total chlorophylls, N, P, Mg, Ca, S, B (p < 0.01). Also, TAA was negatively related with Zn, but this relationship was weaker than that of other relationships with nutrients (p < 0.05). Similarly, AA was negatively associated with chl a & b, carotenoids, total chlorophylls, N, P, Ca, B, Mg, S, and Zn. Additionally, K was inversely correlated with N, P, Mg, S, Zn, Mn, Fe and Cu.

**Table 5:** Correlation coefficients (below diagonal) among all the studied characters of broccoli microgreens with different ascorbic acid treatments.

	TAA (mg 100 g-1 FM)	AA (mg 100 g-1 FM)	chl a	chl b	carotenoids	total chls	N %	P %	K %	Mg %	Ca %	S %	B ppm	Zn ppm	Mn ppm	Fe ppm	Cu ppm
TAA (mg 100 g-1 FM)	1																
AA (mg 100 g-1 FM)	0.95^***^	1															
chl a	-0.37^**^	-0.41^***^	1														
chl b	-0.31^**^	-0.36^**^	0.91^***^	1													
carotenoids	-0.37^**^	-0.39^**^	0.88^***^	0.62^***^	1												
total chls	-0.35^**^	-0.39^**^	0.98^***^	0.97^***^	0.79^***^	1											
N (%)	-0.43^**^	-0.42^**^	0.16^ns^	0.38^*^	-0.12^ns^	0.26^ns^	1										
P (%)	-0.48^**^	-0.46^**^	0.04^ns^	0.20^ns^	-0.14^ns^	0.12^ns^	0.90^***^	1									
K (%)	0.19^ns^	0.26^ns^	-0.02^ns^	-0.10^ns^	0.09^ns^	-0.05^ns^	-0.49^**^	-0.61^***^	1								
Mg (%)	-0.47^**^	-0.38^*^	-0.14^ns^	0.03^ns^	-0.28^ns^	-0.07^ns^	0.75^***^	0.85^***^	-0.39^*^	1							
Ca (%)	-0.49^**^	-0.40^**^	-0.01^ns^	0.15^ns^	-0.14^ns^	0.06^ns^	0.66^***^	0.71^***^	-0.20^ns^	0.86^***^	1						
S (%)	-0.40^**^	-0.32^*^	0.09^ns^	0.21^ns^	-0.06^ns^	0.14^ns^	0.82^***^	0.89^***^	-0.33^*^	0.86^***^	0.78^***^	1					
B (ppm)	-0.52^**^	-0.41^**^	0.18^ns^	0.17^ns^	0.18^ns^	0.18^ns^	0.49^**^	0.54^**^	-0.11^ns^	0.59^***^	0.59^***^	0.63^***^	1				
Zn (ppm)	-0.39^*^	-0.33^*^	0.002^ns^	0.12^ns^	-0.13^ns^	0.06^ns^	0.73^***^	0.82^***^	-0.59^***^	0.81^***^	0.70^***^	0.80^***^	0.42^**^	1			
Mn (ppm)	-0.19^ns^	-0.17^ns^	-0.38^*^	-0.24^ns^	-0.44^**^	-0.33^*^	0.52^**^	0.68^***^	-0.58^***^	0.79^***^	0.63^***^	0.57^***^	0.24^ns^	0.75^***^	1		
Fe (ppm)	0.06^ns^	0.02^ns^	-0.25^ns^	-0.18^ns^	-0.30^ns^	-0.23^ns^	0.41^**^	0.56^***^	-0.76^***^	0.48^**^	0.25^ns^	0.46^**^	0.07^ns^	0.66^***^	0.70^***^	1	
Cu (ppm)	-0.26^ns^	-0.24^ns^	0.002^ns^	0.03^ns^	-0.03^ns^	0.01^ns^	0.52^**^	0.65^***^	-0.63^***^	0.59^***^	0.45^**^	0.65^***^	0.31^*^	0.80^***^	0.58^***^	0.72^***^	1

***, **, and * are significant at p ¾ 0.0001, 0.01, and 0.5 respectively, blue- positive correlation, red- negative correlation and grey/^ns^- non significant, TAA, total ascorbic acid/total Vit C; AA, ascorbic acid; chl a, chlorophyll a; chl b, chlorophyll b; total chls, total chlorophylls; N, nitrogen; P, phosphorus; K, potassium; Mg, magnesium; Ca, calcium; S, sulfur; B, boron; Zn, zinc; Mn, manganese; Fe, iron; Cu, copper.

### 3.3. Differences in EDI and NC of Vit C among the treatments

The highest EDI and NC were found using the treatment with highest ascorbic acid concentration i.e., 0.5% AA followed by 0.25% AA. When the application rate was 0.5% AA, the EDI was 92 mg day^-1^ and the NC was 102.2% (**Table 6**). Lastly, the application of 0.25% AA resulted in an EDI and NC of 47.3 mg/day and 52.5%, respectively.

**Table 6:** Average EDI and NC of Vit C in Broccoli microgreens treated with varying concentrations of ascorbic acid (AA).

Treatments (% AA)	Average Total Vit C (mg/100 g FM)	EDI* of Vit C (mg/day)	NC* (%)
0	38.2	32.5	36.1
0.05	37.0	31.5	34.9
0.1	39.7	33.7	37.5
0.25% AA	55.6	47.3	52.5
0.5% AA	108.2	92.0	102.2

* EDI- Estimated daily intake, NC- Nutrient contribution.

## 4. Discussion

Microgreens are an emerging crop due to their high nutrient density, aesthetic appeal, and unique taste profile. Their usage has been extended from being garnishes to being a regular part of daily diets due to the minimal effort and ease of production in addition to their versatility (Mir et al., 2017). Previous studies have shown the nutrient profile of these microgreens contains high concentrations of mineral nutrients and bioactive compounds that are beneficial to human health (Xiao et al., 2015). Of these, Vit C is an essential micronutrient and antioxidant for humans that can only be obtained through diet. However, daily intake of Vit C is variable and if humans do not consume enough fruits and vegetables in their diets, they may suffer from deficiencies (Locato et al., 2013; Rowe and Carr, 2020). Therefore, sustainable solutions for consistent consumption of nutrient dense plants must be examined when considering human health implications.

Hausen (1935) reported that increasing Vit C is possible in earlier stages of the plant growth cycle through supplemental application of AA. This study further confirms that broccoli microgreens can be biofortified to increase Vit C content with the exogenous application of AA. A previous study by Kathi et al. (2022) showed that arugula microgreens can be biofortified for increased Vit C even at 0.05% AA. These findings contrasted with those of Kathi et al. (2022) in that broccoli microgreens did not show significant increases in TAA and AA at supplemental concentrations of 0.05% in trial 1. However, broccoli microgreens did exhibit significant increases in Vit C at the other AA application rates. More specifically, the 0.1%, 0.25% and 0.5% AA rates had higher concentrations of TAA and AA compared to the lower rate and control. Furthermore, the broccoli microgreen had a 222% increase in Vit C at 0.5% rate in trial 2 whereas arugula microgreens showed 289.4% increase at 0.25% rate. However, 0.5% AA-treated seedlings did not survive during the trial 1 possibly due to environmental differences compared to the trial 2. This further indicates that environmental conditions are important for regulating control of microgreen growth and survival during agronomic biofortification (Di Gioia et al., 2019). Despite the variable effects of supplemental AA between the two microgreens, broccoli microgreens were successfully biofortified to increase Vit C content.

One of the most interesting findings of broccoli microgreens AA supplementation is that they responded differently regarding biomass, chlorophylls, and carotenoids compared to arugula microgreens (Kathi et al., 2022). The broccoli microgreens that received 0.25% AA had an average greater fresh weight (by 53%), dry weight (by 35%), chl a (by 26%), chl b (by 15%), total chls (by 22%) and carotenoids (by 30%) compared to control. Similar results were seen by Noreen et al. (2020) where foliar spray of AA resulted in increased vegetative growth and photosynthetic pigments in barley. This indicates that the broccoli microgreens applied with AA were healthier compared to the control. However, this contrasts with the findings of Kathi et al. (2022) who saw that biomass, chlorophylls, and carotenoids were reduced as AA concentrations increased. The increase in biomass could also be due to the presence of more solutes in the nutrient solution to be absorbed by the plant. This study further supports that different microgreens respond differently in terms of biomass, chlorophylls and carotenoids to supplemental AA application making it an important part of Vit C biofortification studies.

When nutrients in plant tissues were analyzed, a strong inverse correlation was seen between TAA, AA and many nutrients (N, P, Ca, and B). This was also seen with K concentrations, where K concentrations increased, several nutrients and chlorophylls decreased. Overall the concentration of K increased with increased AA in the nutrient solution; the highest K was in the 0.5% treatment, as more KOH was used to buffer the AA. The reduced amounts of nutrients could be due to nutrient antagonism occurring at high concentrations of K with increasing buffer concentrations. Potassium is known to be antagonistic towards P, Mg, Ca, Mn and B when present in large concentrations (Fageria, 2001). But more likely, the proportion of K compared to other nutrients resulted in comparative differences due to greater availability of cations as sum of cations remains almost constant in plant tissues (Fageria, 2001). Yet the nutrient concentrations in plants at the microgreen/seedling stage is highly dictated by cotyledon concentrations of nutrients as at this stage they only have two fully developed cotyledons with the emergence of true leaves (Verlinden, 2019). It should be noted that the concentrations shown in this study were well within the expected levels for broccoli, and no deficiencies were seen. Even though there were patterns that could imply antagonism between different nutrients, the concentration of nutrients found in the biofortified broccoli microgreens in this study were comparable to the results found by Weber (2017). This study also confirms the positive relationship between K and Vit C which is similar to the results reported by Kazemi (2014) where the application of potassium nitrate resulted in increased Vit C. However, for future studies the ascorbic acid could be buffered with something other than KOH to eliminate the possible involvement of K towards the decreasing nutrient content and increasing Vit C.

These results show that there is a positive potential impact for human health and nutrition. The increased total Vit C concentration in biofortified broccoli microgreens resulted in an increased EDI and NC as well. The highest EDI and NC were seen in 0.5% AA treated microgreens with 92 mg/day and 102.2%, respectively. This infers that consumption of FDA reference amount of microgreens resulted in availability of 92 mg of Vit C which contributed 102.2% of daily Vit C requirement. Consumption of these biofortified broccoli microgreens will then result in meeting the daily Vit C requirements of an individual (90 mg) (FDA, 2020).

In conclusion, these biofortified broccoli microgreens offer a greater supply of Vit C compared to that of not biofortified microgreens. Application of AA to broccoli microgreens also improved their biomass, and increased chlorophylls, carotenoids and potassium content.

## 5. Conclusion

This study shows that application of AA through nutrient solutions offers a feasible pathway to increase biomass, chlorophylls, carotenoids, TAA, and AA in broccoli microgreens thereby improving the nutrient composition and dietary value of microgreens. Combined application of AA and KOH also increased K concentrations in addition to the Vit C. While these promising results are positive for the future of biofortification, the assessment of different buffers used in conjunction with AA application should be performed to completely understand the effects of AA alone on the mineral composition of microgreens and eliminate the possible involvement of K. This research documents significant progress towards the agronomic biofortification of Vit C in specialty crops such as microgreens. It also illustrates the importance of investigation in species specific biofortification of vitamins and their relationships to nutrients. Future research exploring the possibility of vitamin C biofortification in different microgreen crops through the AA application offers a promising solution to undernutrition.

## Data availability statement

The raw data supporting the conclusions of this article will be made available by the authors, without undue reservation.

## Author contributions

CS and SK conceived the project. SK carried out experiment, performed analyses and wrote the original draft manuscript. CS and SK performed the statistical analysis. HL, SS, WL, LT assisted with study design and experiments. All authors read and contributed to earlier versions and approved the final version.
